# The novel effectiveness of Tai Chi on cardiopulmonary fitness among stroke patients in the recovery phase: a study protocol for a randomized controlled trial

**DOI:** 10.1186/s13063-021-05565-2

**Published:** 2021-09-13

**Authors:** Tianyang Tan, Yanyan Meng, Xinyu Li, Ruina Bai, Chengchao Wang, Jiaxuan Lyu, Kang Yan, Meng Liu, Chaoyang Zhang, Yulong Wei

**Affiliations:** 1 Beijing University of Chinese Medicine, Beijing, China; 2 China Academy of Chinese Medicine Sciences Xiyuan Hospital, Beijing, China

**Keywords:** Tai Chi, Stroke, Cardiopulmonary fitness, Randomized controlled trial

## Abstract

**Background:**

Stroke is the leading cause of death worldwide. China faces a similar risk of stroke as developed countries because of considerable changes in lifestyle, such as overeating and smoking. Tai Chi is a traditional form of mind-body exercise that has been widely practiced in China for thousands of years. However, there are few studies on the effect of Tai Chi on the cardiopulmonary function of stroke patients in the recovery phase. Therefore, it is necessary to observe the effect of Tai Chi on the cardiorespiratory fitness of patients after stroke.

**Methods:**

This is a parallel-design, two-arm, analyst assessor-blinded, randomized controlled trial. A total of 226 stroke patients in the recovery phase will be recruited and assigned randomly to a control group or Tai Chi group at a 1:1 ratio. The patients in the Tai Chi group will perform the Tai Chi exercise. The patients in the control group will perform walking exercises. Patients in both groups will receive conventional treatments and healthy education. The primary outcomes will be VO_2peak_ and scores on the MOS item short form health survey (SF-36) scale. Secondary outcomes will include vital capacity (VC), ejection fractions (EF), and cardiac output (CO). The assessments of the tests will be performed at three time points (before exercise, at the end of exercise, and 6 weeks after exercise). Adverse events will be recorded faithfully during the study.

**Discussion:**

If the results are positive, this study will contribute to the establishment of further guided Tai Chi rehabilitation programs.

**Trial registration:**

Chinese Clinical Trial Registry ChiCTR2000034719. Registered on 16 July 2020.

## Administrative information

The order of the items has been modified to group similar items (see http://www.equator-network.org/reporting-guidelines/spirit-2013-statement-defining-standard-protocol-items-for-clinical-trials/).

**Table:** 

Title {1}	The novel effectiveness of Tai Chi on cardiopulmonary fitness of stroke patients in the recovery phase: a study protocol for a randomized controlled trial
Trial registration {2a and 2b}.	ChiCTR2000034719[Chinese Clinical Trial Registry] http://www.chictr.org.cn/edit.aspx?pid=56653&htm=4[registered on 18-07-2020]
Protocol version {3}	This protocol is the first version 1, which was approved on 16 July 2020
Funding {4}	This research is funded by National Key Research and Development Plan of China(2019YFC1710303)
Author details {5a}	Tianyang Tan: Beijing University of Chinese Medicine Xinyu Li: Beijing University of Chinese Medicine Ruina Bai: China Academy of Chinese Medicine Sciences Xiyuan Hospital Chengchao Wang: Beijing University of Chinese Medicine Jiaxuan Lv: Beijing University of Chinese Medicine Kang Yan: Beijing University of Chinese Medicine Meng Liu: Beijing University of Chinese Medicine Chaoyang Zhang: Beijing University of Chinese Medicine Yanyan Meng*: Beijing University of Chinese Medicine Yulong Wei*: Beijing University of Chinese Medicine
Name and contact information for the trial sponsor {5b}	Investigator initiated clinical trial;Yulong Wei wyl_5128@163.com
Role of sponsor {5c}	This is an investigator initiated clinical trial. Therefore, the funders played no role in the design of the study and collection, analysis, and interpretation of data and in writing the manuscript.

## Introduction

### Background and rationale {6a}

Stroke is the leading cause of death worldwide [1]. Accordingly, stroke has become the top cause of death and disability in China [2], reducing the quality of life and increasing the economic burden on families and society.

After the occurrence of stroke, prolonged muscle weakness and dystonia [3] promote deformities of bones, joints, and ligaments [4], thereby causing balance and motor disorders. In addition, 44% to 74% of patients [5] still have varying degrees of cognitive impairment after a stroke, and 25% to 74% of patients suffered from psychological disorders such as depression [6]. Balance ability [7], motor ability, cognition [8], and mood [9] are positively correlated with cardiopulmonary fitness (CRF), which influences the survival rate after stroke [10]. Moreover, the time period after a stroke ranges from 10 days to 8 years, suggesting that the decline in cardiorespiratory fitness may persist for years after the stroke. CRF reflects [11] the ability of the circulatory and respiratory systems to supply oxygen to skeletal muscles during sustained, moderate-to-intense exercise physical activity. Poor cardiopulmonary fitness, due to low exercise for 1–3 years [12] after stroke, may be a secondary factor limiting rehabilitation [13]. Conversely, cardiopulmonary training and consequent improvements in cardiopulmonary fitness increase motor function, the frontal gray [14] (which is associated with emotion [15]) and hippocampal volumes [16] (which influence cognition [17]).

Traditionally, stroke rehabilitation has focused on primary neurological impairments, including muscle weakness and loss of coordination. The intensity of physical or occupational therapy activities is not sufficient to cause cardiopulmonary training effects [18]. Tai Chi is a traditional form of mind-body exercise that has been widely practiced in China for thousands of years. The fitness of this exercise is believed to facilitate the flow of qi through the body, thereby producing health benefits. There are various styles of Tai Chi, including Yang-style (24-form) and Sun-style (21-form) styles. Strong evidence has shown that Tai Chi improves or maintains balance ability in patients with Parkinson's disease [19]. In addition, Tai Chi has been shown to play a positive role in CRF. A study showed that Tai Chi significantly [20] reduced elderly obese people’s vital capacity and maximal oxygen uptake. Tai Chi improves [21] cardiopulmonary fitness parameters, including forced expiratory volume in 1 s (FEV1) and the 6-minute walk test (6MWT), in patients with chronic obstructive pulmonary disease (COPD). Moreover, Tai Chi has been widely practiced for stroke rehabilitation. Specifically, prior studies show that Tai Chi training may constitute a viable intervention not only to improve balance [22] and quality of life [23] in stroke survivors but also to support stroke prevention [24].

However, the effects of Tai Chi on specific stimulation actions for cardiopulmonary fitness have rarely been studied. Because 24-form Tai Chi requires higher strength, balance, and coordination of the upper and lower limbs, it is challenging for patients in the stroke recovery period to achieve psychosomatic exercise training effects. Therefore, six suitable movements were selected from the 24 forms of Tai Chi prepared by the General Administration of Sport of China in 1956. The “Six Forms of Tai Chi” are Commencing Form, Wave Hands Like Clouds, Repulse Monkey, Part the Wild Horse's Mane on Both Side, Hold the Lute, and Closing Form. We adapted six movements according to the theories of normal human anatomy, traditional Chinese medicine (TCM) meridians and collaterals, TCM Qigong, etc., to produce specific effects on cardiopulmonary fitness. To date, no studies have clarified the effect of “Six Forms of Tai Chi” training on the cardiopulmonary fitness of patients after stroke.

### Objectives {7}

Based on previous literature and training experience, our hypothesis is that “Six Forms of Tai Chi” training safely improves the cardiopulmonary fitness of stroke patients in the recovery phase.

### Trial design {8}

This study will be a multicenter, single blind (assessor and statistician), 19-week, randomized parallel control group trial. Patients will be allocated in a 1:1 ratio into the control group or Tai Chi group.

## Methods: Participants, interventions, and outcomes

### Study setting {9}

**Fig. 1:**
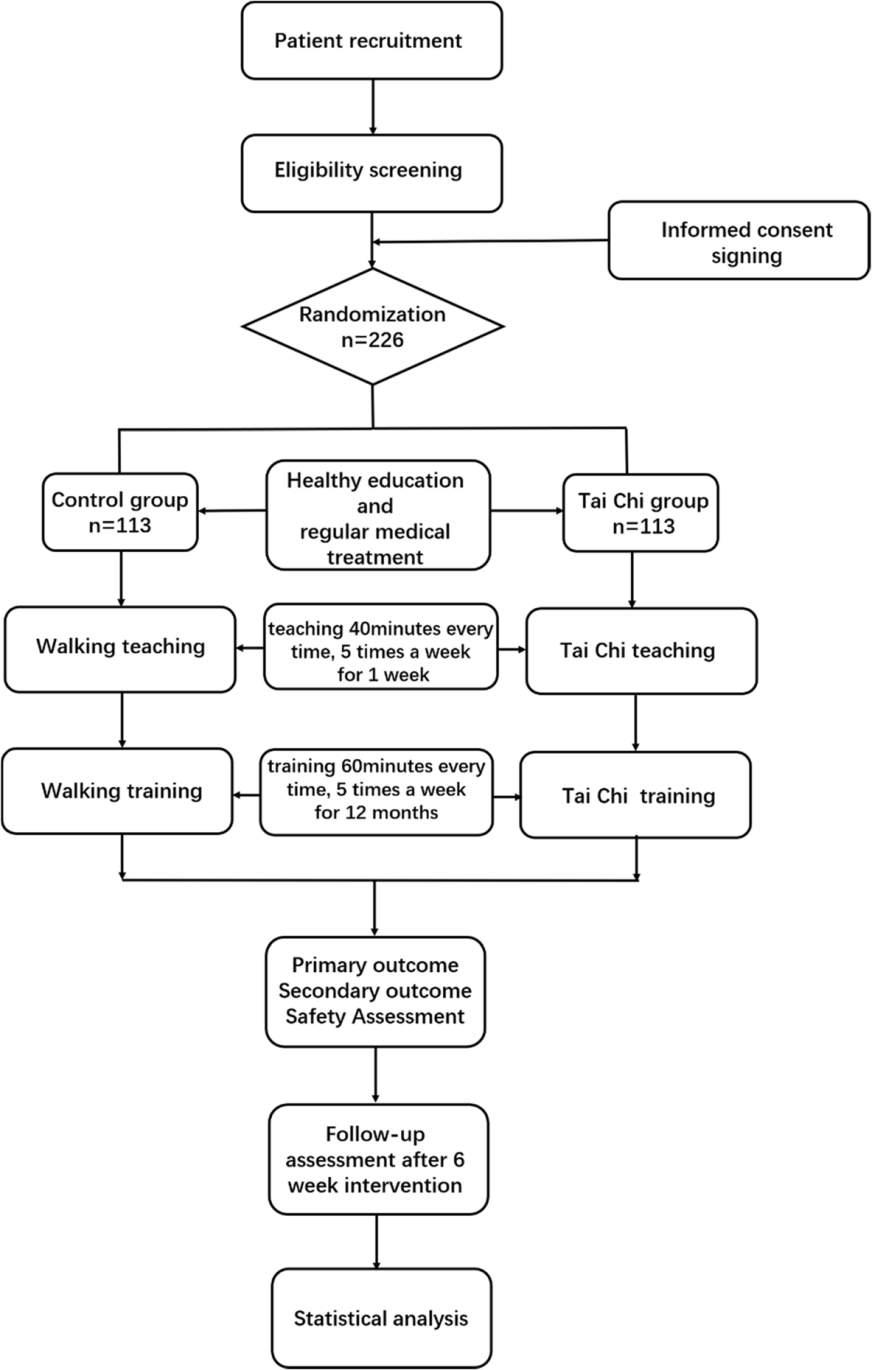
Flow chart of the trial

The patients will be recruited from the Xi Yuan Hospital, Beijing Da Xing District of Integrated Traditional Chinese and Western Medicine, Hebei Cang Zhou Hospital of Integrated Traditional Chinese and Western Medicine, Fujian University of Chinese Medicine, Henan Provincial People’s Hospital, The Third People’s Hospital of Zhengzhou, and Henan Provincial Hospital of TCM. Patients will be considered for inclusion if they meet the criteria defined below. The flow chart is shown in Fig. 1.

### Eligibility criteria {10}

Inclusion criteria (at the outpatient clinic or inpatient)

Patients must meet the following criteria to be eligible for the study:

1. Key Points for the Diagnosis of Various Major Cerebrovascular Diseases in China 2019.
2. 2 weeks to 6 months after onset of stroke.
3. Between 18 and 75 years of age.
4. Berg balance ability scale > 40 points.
5. Patients or their guardians must sign an informed consent form.

Exclusion criteria (at the outpatient clinic or inpatient)

1. Subarachnoid hemorrhage.
2. Severe dysfunction or complications of liver and kidney.
3. Peripheral facial paralysis, visual impairment, aphasia, and mental disorders that prevent the patient from completing the study.
4. Recent acute coronary syndrome, including myocardial infarction or unstable angina (within 4 weeks).
5. Patients with chronic obstructive pulmonary disease.
6. Systolic blood pressure > 180 mmHg, diastolic blood pressure > 100 mmHg, and resting heart rate > 120 beats/min.
7. Patients who participated in Tai Chi training within 3 months.
8. MOCA scale ≤26 points.
9. The 6-minute walking test was completed with a BORG score of less than 3 or greater than 7.

### Who will take informed consent? {26a}

Stroke patients in the recovery phase will be screened for eligibility to participate in this study based on the abovementioned criteria. After the patient has been assessed as eligible by the lead physician, he/she will receive initial study information. After at least 2 weeks of reflection, stroke patients in the recovery phase will be invited to meet with the research physician to discuss any remaining questions and sign the informed consent form.

### Additional consent provisions for collection and use of participant data and biological specimens {26b}

A total of 226 stroke patients will be asked to supply 10 ml of blood. These blood samples of patients will be drawn at baseline and at 13 and 19 months. Regardless of treatment allocation, patients will be made aware this part of the trial on the informed consent form.

## Interventions

### Explanation for the choice of comparators {6b}

According to the Physical Activity and Exercise Recommendations for Stroke Survivors, the control group will receive walking training with standard care and health education interventions.

### Intervention description {11a}

The participants in the Tai Chi group will undergo “Six Forms of Tai Chi” training in the garden of the hospital or campus gymnasium, with 60-minute sessions occurring five times a week for 3 months. Before formal training, Tai Chi teaching will be held for 1 week, including lessons on the knowledge of Tai Chi and the six movement standards of Tai Chi. Formal training will be conducted after all participants have similar assessment scores. According to the Physical Activity and Exercise Recommendations for Stroke Survivors, each session will include 40 min of Tai Chi training plus 10 minutes of warm-up and cool down, and the intensity will be 55–80% heart rate (HR) max. The six Tai Chi movements will be adapted by an expert panel from the Yang Short-form style that is based on the General Administration of Sport of China. The six movement main points are as shown in Table 1.

**Table 1:** Chart of six movement main points

Number	Name	Main point	Breathing
Form 1	Commencing form	Raise palm	Inhale
		Down push both palms	Exhale
Form 2	Wave hands like clouds	Back pull on shoulders and elbows	Inhale
		Palm down	Exhale
Form 3	Repulse monkey	Turn and hold palm up	Inhale
		Return to palm and look forward	Exhale
Form 4	Part the wild horse’s mane on both side	Push palm in bow step	Inhale
		Exhale and settle palms	Exhale
Form 5	Hold the lute	Suction drives the shoulder up	Inhale
		The exhale pulls the shoulders back down	Exhale
Form 6	Closing form	Raised both arms	Inhale
		Down push both palms	Exhale

The six movements will be combined with abdominal breathing and will focus on cardiopulmonary fitness in stroke patients in the recovery phase. At the end of each training, the participants will be required to complete a “Tai Chi training log” to evaluate the training quality and compliance. Tai Chi training will be provided by Tai Chi coaches who achieved similar results in the qualification examination. All participants in the Tai Chi group will receive regular medical treatment and health education.

According to the Physical Activity and Exercise Recommendations for Stroke Survivors, the participants will conduct walking training. Before walking training, walking teaching will be held for 1 week. The training time, frequency and intensity were consistent with those of the Tai Chi group. Each session will include 40 min of walking exercise plus 10 min of warm-up and cool down. All participants in the control group will also receive regular medical treatment and health education as the Tai Chi group.

### Criteria for discontinuing or modifying allocated interventions {11b}

If the study meets any of the following criteria, the interventions will prematurely end. Patients can leave the study at any time for any reason if they wish to do so and will not face any consequences. Patient participation in this study can be ended by the investigator if the patient is uncooperative and/or does not attend study visits. During the study, patients with low adherence can affect the authenticity of the results. Interventions will be discontinued if there are abundant adverse events (AEs) or serious adverse events (SAEs).

### Strategies to improve adherence to interventions {11c}

To improve training compliance, the coach will constantly emphasize the significance and benefits of training for participants. After each training session, participants will complete the self-feeling scale to report their feelings in a timely manner. The coach will regularly conduct out WeChat, telephone return visits, and time training.

### Relevant concomitant care permitted or prohibited during the trial {11d}

Conventional drug treatment (antihyperglycemic, antihypertensive, etc.) will be allowed. Patients’ medication status will be recorded continuously. The control group will not be allowed to take Tai Chi training until the end of the 6-week follow-up.

### Provisions for post-trial care {30}

This study will not include any ancillary or posttrial care. There is no compensation budget item for this study.

### Outcomes {12}

#### Primary outcomes

1. VO_2Peak_: Peak oxygen uptake (VO_2Peak_) is a highly reproducible objective indicator of cardiopulmonary fitness by the cardiopulmonary exercise test (CPET), which reflects the maximum oxygen uptake achieved by the participant through maximum effort. Studies have shown that VO_2Peak_ is positively correlated with regional cerebral blood flow [25]. Measurement of VO_2Peak_ by CPET is generally recognized as the gold standard of cardiopulmonary capacity [26].
2. SF-36: The SF-36 is a general health status parameter designed for population surveys or health assessments. It contains both physical health content (physical fitness, role-physical, body pain, general health) and mental health content (vitality, social fitness, role emotional, mental health). In this study, the SF-36 scale will be used to assess the effects of Tai Chi on the physical and mental health of stroke patients.

#### Secondary Outcomes

1. Cardiopulmonary fitness-related indicators: vital capacity (VC), oxygen saturation (SPO_2_), heart rate variability (HRV), cardiac output (CO), ejection fractions (EF), maximal voluntary ventilation (MVV), etc.
2. Safety Assessment: Adverse events (AEs) will be observed during the study period, and the degree of correlation with the study measures will be assessed according to a 5-level standard: ① absolutely related, ② probably related, ③ may be related, ④ may be not related, and ⑤ absolutely unrelated. Regardless of whether adverse events were associated with the study, researchers recorded details on the case report form (CRF), including time, symptoms, adverse reactions and seizure frequency, continuous termination time, laboratory examination indexes, treatment methods, and results.

The researchers will be required to report any serious adverse event (SAE) to the research group within 24 hor no later than the second working day. Researchers will sign and date the report and recorded the source when, how, and to whom serious adverse events were reported.

The formula is as follows: Incidence of adverse events = (①+②+③)/Total actual case.

#### Other outcomes

(1) Molecular mechanism: Surfactant protein D (SP-D) and N-terminal pro-brain natriuretic peptide (NT-proBNP). Serum SP-D levels are considered to be a disease marker value for human lung diseases, including interstitial lung diseases (ILDs), COPD, and asthma [27]. Serum BNP levels may [28] help identify right heart failure or pump thrombosis/malfunction. Many clinical trials have shown that Tai Chi training effectively reduces the level of brain natriuretic peptide in patients with chronic heart failure [29] and myocardial infarction.

(2) Berg balance scale: Disturbance of balance is the most common cause of disability in stroke patients. The Berg balance scale has been widely used to evaluate poststroke patients’ abilities [30]. The scale consists of 14 items, each of which is divided into 5 grades from 0 to 4, with a full score of 56. Higher scores indicate better balance.

### Participant timeline {13}

The participant timeline is shown in Table 2.

**Table 2:**
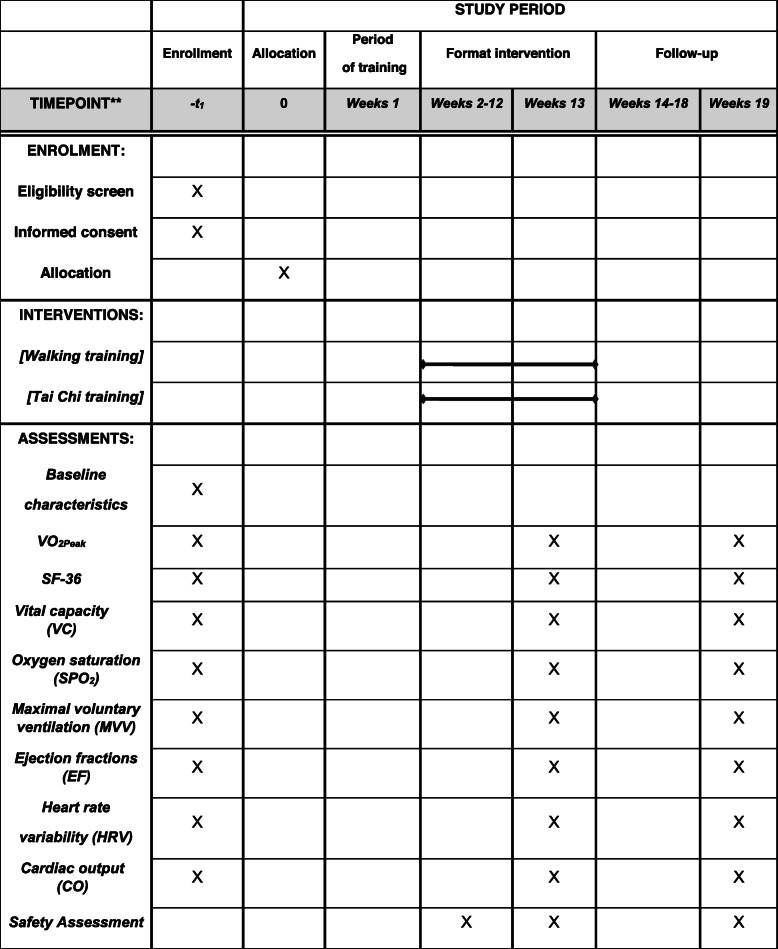
Example template of recommended content for the schedule of enrollment, interventions, and assessments

### Sample size {14}

Sample size estimation in this trial is based on the expected improvement of VO_2Peak_ in 12 weeks of Tai Chi training in patients with heart failure [31]. After 12 weeks of intervention, the VO_2Peak_ of the aerobic exercise group and Tai Chi group was respectively 13.0 ± 4 ml/kg/min and 15.2±6 ml/kg/min. We set *α* = 0.05, *β* = 0.10. The sample size will be estimated according to the estimation formula:

$$ {n}_1={n}_2=2{\left[\frac{\left({t}_{2a}+{t}_{2\beta}\right)\;s}{\delta}\right]}^2 $$

It is estimated that a sample size of 94 participants per group will be required. Considering a 20% dropout and exit rate, we will recruit 226 participants, with 113 participants in each group.

### Recruitment {15}

The patients will be recruited from the Xi Yuan Hospital, Beijing Da Xing District of Integrated Traditional Chinese and Western Medicine, Hebei Cang Zhou Hospital of Integrated Traditional Chinese and Western Medicine, Fujian University of Chinese Medicine, Henan Provincial People’s Hospital, The Third People’s Hospital of Zhengzhou, and Henan Provincial Hospital of TCM.

## Assignment of interventions: allocation

### Sequence generation {16a}

Participants will be randomly assigned to the control group or Tai Chi group (1:1) via block randomization at baseline using a central stochastic system.

### Concealment mechanism {16b}

Allocation is not concealed and will be revealed to both the participants and the researcher upon randomization.

### Implementation {16c}

Each hospital is equipped with a random staff. The account and password of the random staff are given to enter the random system (http://121.229.17.89:9080/IWRS/). Patients will receive a unique random number from random staff.

## Assignment of interventions: blinding

### Who will be blinded {17a}

In this study, it will be impossible to blind the participants and Tai Chi coaches. To minimize bias, study personnel, such as researchers who perform relevant tests and outcome evaluators, will not have permission of a central stochastic system. Group A will be used to identify the control group, and group B will be used to identify the Tai Chi group, thus blinding the statisticians and outcome evaluators.

### Procedure for unblinding if needed {17b}

When statisticians complete the work of data management and data analysis, the random allocation will be uncovered.

## Data collection and management

### Plans for assessment and collection of outcomes {18a}

Baseline characteristic data will be collected from electronic personal medical records and recorded with an electronic case report form (eCRF) using Xi Yuan Hospital EDC. All serum samples will be subjected to laboratory tests. All data acquired during the study will be anonymized and saved in a study folder on our protected research server.

### Plans to promote participant retention and complete follow-up {18b}

The importance of completion of all trials will be informed. The participants will be able to stop at any time by themselves without giving a reason. At each point in the trial, all patients will be prompted to complete the assessments by telephone or WeChat.

### Data management {19}

The evaluators will record the data on the paper case report form, and the complete test report will be attached. The data entry personnel of each hospital will be input into the eCRF once a week and managed by independent organization.

### Confidentiality {27}

To protect information confidentiality, participants will be identified only by acronyms, and their real names will not appear on any reports during the study. Each hospital has an exclusive data administrator who will be the only person with access to the eCRF.

### Plans for collection, laboratory evaluation, and storage of biological specimens for genetic or molecular analysis in this trial/future use {33}

At baseline, week 12 and week 19, 10 ml blood from participants will be collected, and blood will be isolated at 3000 r/min for 10 min. After the serum entered the frozen pipe, it was stored in a refrigerator at − 80 °C. Independent organization will test serum biochemicals uniformly.

## Statistical methods

### Statistical methods for primary and secondary outcomes {20a}

Statistical analysis will be performed by statisticians who come from the third term using SPSS 20.0 software for Windows. Categorical variables will be expressed in terms of frequency or percentage, and continuous variables will be expressed in terms of mean and standard deviation. Primary and secondary outcome data collected after 13 weeks and 19 weeks will be compared with baseline data, respectively. For normally distributed variables, a *t* test will be used for statistical comparisons between groups. If it is nonnormal, then a nonparametric test is used. *P* values less than 0.05 will be considered statistically significant. To verify that the results are consistent, the primary outcome (change in VO_2Peak_ between baseline and 19 weeks) will be compared between the two groups using intention-to-treat (ITT) and per-protocol subject analysis (PPS). The Pearson correlation or Spearman correlation measures the correlation between two variables.

### Interim analyses {21b}

There are no interim analyses planned.

### Methods for additional analyses (e.g., subgroup analyses) {20b}

For the primary and secondary outcomes, we will perform subgroup analyses according to age.

### Methods in analysis to handle protocol non-adherence and any statistical methods to handle missing data {20c}

Missing data patterns will be analyzed by means of missing value analyses.

### Plans to give access to the full protocol, participant-level data, and statistical code {31c}

There are no plans to give access to these data or codes.

## Oversight and monitoring

### Composition of the coordinating center and trial steering committee {5d}

This is a single-center study performed and coordinated at the Beijing University of Chinese Medicine. Day to day support for the trial will be provided by:

1. Principle investigator: supervise the trial and medical responsibility of the patients.
2. Study coordinator: trial registration, coordinate study visits.
3. Head coach of Tai Chi: Establish the “Six Forms of Tai Chi” criteria and train the coach of each center.

The study team will meet every 3 weeks. There will not be a trial steering committee or stakeholder or public involvement group.

### Composition of the data monitoring committee, its role and reporting structure {21a}

Not applicable. A data monitoring committee is not needed, as risks are expected to be minimal.

### Adverse event reporting and harms {22}

During the entire study, minor or moderate adverse events will be recorded faithfully in CRF. The coordinating center will conduct a comprehensive analysis and assessment of the occurrence of adverse reactions. Serious adverse events occurring during the study period, regardless of whether they are related to the study-related examination and treatment, will be reported to the central research group within 24 h. Study personnel will complete the registration form of serious adverse events, give symptomatic treatment and actively address the event.

### Frequency and plans for auditing trial conduct {23}

The auditing trial will be conducted online for 2 weeks. The audit includes data (completeness, accuracy, clarity) and quality of intervention (attendance, exercise intensity, side effect of exercise). These contents will be sent to the research group in the form of pictures, videos, and forms, and the research group will give feedback timely. The site audit will be conducted per 8–10 weeks to ensure a more realistic auditing of the trial details.

### Plans for communicating important protocol amendments to relevant parties (e.g., trial participants, ethical committees) {25}

Any modifications (the principal investigator, clinical research protocol, informed consent, recruitment materials, etc.) will be submitted to the Ethics Committee of Beijing University of Traditional Chinese Medicine for review and approval of amendments.

### Dissemination plans {31a}

The results of this study will be published entirely in international peer-reviewed journals. Both positive and negative results will be reported. If participants choose to receive study-level results, they will receive a nonprofessional summary of the results.

## Discussion

Modern medicine believes that cardiopulmonary fitness has a close relationship with blood perfusion and stroke rehabilitation. Because stroke often leads to a sedentary lifestyle [32], stroke patients [33] have shown a 50% reduction in CRF compared to their peers, and the VO_2Peak_ value was 8–22 ml/kg/min, which was equivalent to 26–87% of the VO_2Peak_ value of matched healthy individuals [34]. Marked reduced cardiopulmonary fitness [35] is often accompanied by weakness of the breathing muscles, including the diaphragm, intercostal muscles, and abdominal muscles. These breathing muscles contribute to balance ability, which is a major cause of impairment for patients with stroke via diaphragmatic contraction and by increasing intra-abdominal pressure. VO_2Peak_, as a gold indicator of cardiopulmonary fitness [7], is associated with an increased volume of gray matter in the anterior cingulate inferior frontal gyrus [36]. Cardiopulmonary fitness may be a basis for functional recovery after stroke.

“Heart dominating blood and vessels” and “Lung dominating breath” are from the Traditional Chinese Medical. With moderate exercise [37], qi and blood will be mobilized when practicing Tai Chi. The coordination of the heart and lung promotes the movement of qi and blood. Some studies show that regular Tai Chi training is proven to affect heart rate in cardiovascular risk factors in elderly individuals [38] or community-dwelling older individuals [39]. Various biomarkers, such as endothelin, brain natriuretic peptide, and tumor necrosis factor, are modulated according to Tai Chi mechanistic studies. Although there is a lack of Tai Chi on cardiopulmonary fitness in stroke patients in the recovery phase, it can be inferred from the literature that Tai Chi has the potential to improve the cardiopulmonary fitness of stroke patients. However, emerging evidence has focused on the effects of Tai Chi on balance, motor function, and depression in poststroke patients. Few researchers have been conducted on the effects of Tai Chi on cardiopulmonary fitness during stroke recovery. Therefore, it is necessary to further study the effect of Tai Chi on the cardiorespiratory fitness of patients after stroke. CEPT is a noninvasive way to obtain information about a patient’s cardiorespiratory fitness. CEPT integrates circulatory, respiratory, and metabolic systems, thus allowing researchers to observe the function of the cardiovascular and respiratory systems simultaneously. Hence, CEPT is selected for this study.

In this innovative study, we are conducting the first randomized control trial of the “Six Forms of Tai Chi” versus walking training, which has documented guidelines for cardiopulmonary training in a large poststroke population. If the results are positive, this study will contribute to the establishment of further guided Tai Chi rehabilitation programs.

## Trial status

This protocol is the first version 1, which was approved on 16 July 2020.

The trial was started on 1 September 2020. We hope to achieve our research objectives by December 2021.

## Data Availability

To protect the privacy of participants, the data sets generated or analyzed in this study have not been made public, but can be obtained from the corresponding author if reasonably requested

## Abbreviations

CRF: Cardiopulmonary fitness
FEV1: Forced expiratory volume in 1 s
6MWD: 6-minute walk test
COPD: Chronic obstructive pulmonary disease
TCM: Traditional Chinese medicine
HR: Heart rate
AE: Adverse events
SAE: Serious adverse events
VO_2Peak_: Peak oxygen uptake
CPET: Cardiopulmonary exercise test
VC: Vital capacity
SPO_2_: Oxygen saturation
HRV: Heart rate variability
CO: Cardiac output
EF: Ejection fractions
MVV: Maximal voluntary ventilation
NT-proBNP: N-terminal pro-brain natriuretic peptide
ILD: Interstitial lung diseases
SP-D: Surfactant protein D
eCRF: Electronic case report form
